# Simultaneous ipsilateral nephrectomy during kidney transplantation in autosomal dominant polycystic kidney disease: a matched pair analysis of 193 consecutive cases

**DOI:** 10.1007/s00423-020-01939-3

**Published:** 2020-07-23

**Authors:** Bernd Martin Jänigen, Johann Hempel, Philipp Holzner, Johanna Schneider, Stefan Fichtner-Feigl, Oliver Thomusch, Hannes Neeff, Przemyslaw Pisarski, Torben Glatz

**Affiliations:** 1 Faculty of Medicine, Department of General and Digestive Surgery, Section of Transplant Surgery, Medical Center, University of Freiburg, Freiburg im Breisgau, Germany; 2 Faculty of Medicine Department of Medicine IV, Medical Center, University of Freiburg, Freiburg im Breisgau, Germany; 3 Department of Surgery, Marien Hospital Herne, Ruhr-University Bochum, Herne, Germany

**Keywords:** Simultaneous unilateral nephrectomy, Renal transplantation, Polycystic kidney disease, ADPKD

## Abstract

**Background:**

In end-stage renal transplant recipients with autosomal-dominant polycystic kidney disease (ADPKD), the imperative, optimal timing, and technique of native nephrectomy remains under discussion. The Freiburg Transplant Center routinely performs a simultaneous ipsilateral nephrectomy.

**Methods:**

From April 1998 to May 2017, we retrospectively analyzed 193 consecutive ADPKD recipients, receiving per protocol simultaneous ipsilateral nephrectomy and compared morbidity, mortality, and outcome with 193 non-ADPKD recipients of a matched pair control.

**Results:**

The incidence of surgical complications was similar with respect to severe medical, surgical, urological, vascular, and wound-related complications as well as reoperation rates and 30-day mortality. Intraoperative blood transfusions were required more often in the ADPKD (22.8%) compared with the control group (6.7%; *p* < 0.0001). Early postoperative urinary tract infections occurred more frequent (ADPKD 40.4%/control 29.0%; *p* = 0.0246). Time of surgery was prolonged by 30 min (ADPKD 169 min; 95%CI 159.8–175.6 min/control 139 min; 95%CI 131.4–145.0 min; *p* < 0.0001). One-year patient (ADPKD 96.4%/control 95.8%; *p* = 0.6537) and death-censored graft survival (ADPKD 94.8%/control 93.7%; *p* = 0.5479) were comparable between both groups.

**Conclusions:**

With respect to morbidity and mortality, per protocol, simultaneous native nephrectomy is a safe procedure. Especially in asymptomatic ADPKD KTx recipients, the number of total operations can be reduced and residual diuresis preserved up until transplantation. In living donation, even preemptive transplantation is possible.

## Introduction

The risk of end-stage renal disease (ESRD) in ADPKD patients is age-related. By the age of 60 years, about 50% of patients require dialysis [1]. This group represents about 10–15% of patients on dialysis [2] and approximately 10% of all renal transplant recipients [3, 4]. Other treatment options, targeting the underlying pathophysiological mechanisms in order to preserve kidney function are currently being evaluated [5]. Apart from renal failure patients with ADPKD most commonly suffer from recurrent urinary tract infection (UTI), cyst infection, refractory pain, hematuria, and digestive disturbances due to space constraints. Presently, therapy for ADPKD centers on the renal-related morbidity, which affects quality of life and life expectancy [3, 6, 7]. Commonly, surgical intervention is required to deal with these severe ADPKD-related complications and often native nephrectomy is the only therapeutic option. With regard to a renal transplantation, space restrictions by large native kidneys are a serious problem. In asymptomatic patients, a preemptive renal transplantation is controversial with respect to timing and procedural issues. Simultaneous or post-transplant nephrectomy appears beneficial by avoiding pre-transplant dialysis [8]. In waitlist dialysis recipients, with preserved urinary excretion, diuresis is not compromised by nephrectomy. Yet concerns about placing renal graft function at risk by adding simultaneous nephrectomy to the procedure in asymptomatic recipients or by post-transplant ipsilateral nephrectomy for post-KTx symptoms caused by the remaining kidney have to be addressed. In summary, the question remains: Are we putting patients at risk for surgical complications by the nephrectomy and a prolonged operation time when pursuing a per protocol simultaneous approach.

The aim of this retrospective study is to analyze whether simultaneous nephrectomy of a polycystic kidney during KTx as a standard procedure is commendable or accompanied by a higher risk of surgical complications and negative effects on short-term patient and graft survival compared with kidney transplantation alone.

## Materials and methods

### Study design and patients

From the late 1990s, per protocol simultaneous unilateral native nephrectomy in all cases of kidney transplantation for ADPKD became the policy at our Transplant Center. From April 1998 to May 2017, a total of 1595 renal transplantations in adult recipients were performed in Freiburg. Forty-two KTx recipients with an incomplete data set and 29 en-bloc Ktx recipients were excluded. In the remaining group of 1524 patients, we identified 193 recipients with ADPKD receiving simultaneous ipsilateral nephrectomy during renal transplant procedure (ADPKD group). Subsequently, the residual 1331 KTx recipients were screened for patients receiving transplantation in combination with simultaneous nephrectomy of a former renal graft or native non-polycystic kidney. A total of 106 recipients were identified and excluded from the analysis, leaving 1225 potential candidates for the control group.

**Fig. 1:**
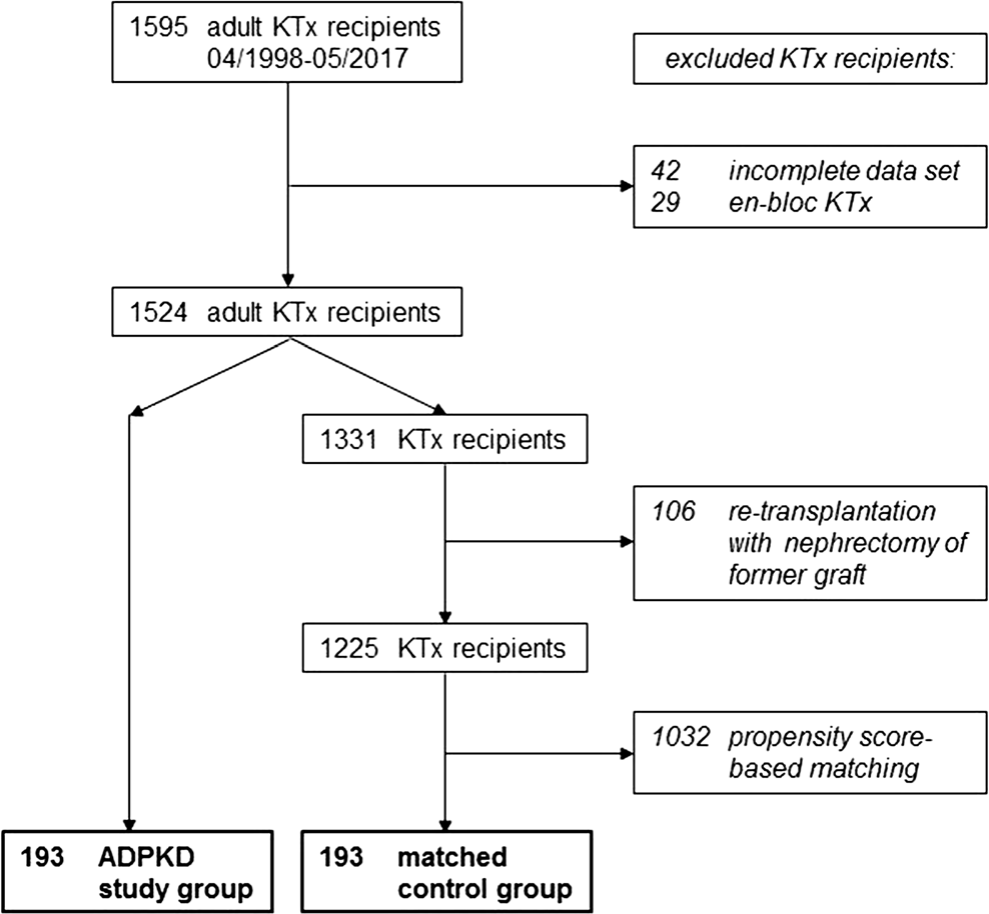
Study profile. The study compares 193 consecutive ADPKD renal transplant recipients receiving simultaneous ipsilateral nephrectomy with a propensity score-based matched pair control group

The control group was composed of 193 matched patients. The propensity score-based matching was performed with M&M: propensity score matching by nearest neighbor method using MatchIt package of R software (www.r-project.org), as described [9]. The following parameters were included (a) recipient and donor age, (b) time on dialysis, (c) type of transplantation, (d) cold ischemic time, (e) number of transplantation, (f) panel reactive antibodies, (g) HLA-mismatches, and (i) year of transplantation (Fig. 1; Table 1). In spite of scanning the database prior to the matching process, 3 (1.6%) KTx recipients remained in the control group, who underwent a simultaneous non-polycystic native (*n* = 2, 1.0%) or a nephrectomy of a former renal graft (*n* = 1, 0.5%).

**Table 1:** Preoperative baseline characteristics of study population

		ADPKD group (*n* = 193)	Matched control group (*n* = 193)	*p* value
*Donor characteristics*				
*Donor age (years)		53.0 (95% CI 51.0–56.0)	57.0 (95% CI 54.0–60.0)	0.1744
Donor gender (female/male)		98 (50.8%)/95 (49.2%)	86 (44.6%)/107 (55.4%)	0.2623
Donor BMI (kg/m^2^)		25.6 (95% CI 24.8–26.1)	25.3 (95% CI 24.7–25.9)	0.3551
*Recipient characteristics*				
*Recipient age (years)		54.6 (95% CI 53.0–56.2)	55.7 (95% CI 53.0–59.1)	0.7703
Recipient gender (female/male)		88 (45.6%)/105 (54.4%)	61 (31.6%)/132 (68.4%)	0.0065
Recipient BMI (kg/m^2^)		25.1 (95% CI 24.6–26.2)	25.2 (95% CI 24.7–25.9)	0.9309
ASA status recipient (in %)	1 2 3 4	0 (0.0%) 63 (32.6%) 122 (63.2%) 8 (4.2%)	1 (0.5%) 54 (28.0%) 130 (67.4%) 8 (4.2%)	1.0000 0.3757 0.4543 1.0000
*Time on dialysis (in d)		964 (95% CI 577.0–1345.4)	909 (95% CI 719.4–1229.7)	0.8064
*Transplant characteristics*				
*Type of transplantation (in %)	Cadaveric ABOc (living) ABOi (living)	96 (49.7%) 75 (38.9%) 22 (11.4%)	94 (48.7%) 76 (39.4%) 23 (11.9%)	0.9189 1.0000 1.0000
*Cold ischemic time (in min.)		328.0 (95% CI 216.0–436.6)	278.0 (95% CI 181.4–435.4)	0.1088
*Year of transplantation		2008 (95% CI 2007–2009)	2008 (95% 2007–2009)	0.2102
*Immunological characteristics*				
*No. of transplantation (in %)	1st 2nd 3rd	187 (96.9%) 6 (3.1%) 0 (0.0%)	184 (95.3%) 7 (3.6%) 2 (1.0%)	0.5999 1.0000 0.4987
*Panel reactive antibodies (in %)		0.0 (95% CI 0.0–0.0)	0.0 (95% CI 0.0–0.0)	0.6927
*HLA-Mismatches (no. of mismatches)		3.0 (95% CI 3.0–4.0)	3.0 (95% CI 3.0–3.0)	0.2351

Data are *n* (%) or median ± 95% CI. *p* values are estimated with Mann-Whitney U test for calculated data, and 2-tailed Fisher’s exact test for categorical data

*propensity score-based match criteria

### Transplant procedure, nephrectomy, postoperative care, and follow-up

All recipients were placed in supine position. The kidney transplantation was performed in the established technique usually to the right iliac fossa. Lymphatic tissue covering the blood vessels was removed thoroughly, keeping the vascular implantation sites as small as possible. In the case of simultaneous ipsilateral nephrectomy, the hockey stick incision was expanded up to the coastal arch. The access for the nephrectomy was strictly kept retroperitoneal. During the removal of the polycystic kidney, main vessels were closed with non-resorbable sutures and effective hemostasis of the retroperitoneal space by electric coagulation was routinely carried out. The technique of kidney transplantation was not altered from standard protocol for ADPKD patients. Grafts were surface cooled during anastomosis time. Each patient received an intraoperative antibiotic single-shot with 1.5 g cefuroxime or 2 g cephzoline. For thromboembolic prophylaxis recipients received a daily dose of 12,500 units of heparin starting 6 h after termination of surgery. All procedures were performed by an experienced transplant team involving only five surgeons over the years. Double-J stents were routinely inserted and removed after 10–15 days. Wound drainage was removed once secretion was less than 100 ml per day (usually between day 4 and 8). All recipients were hospitalized for approximately 2 weeks on our transplant ward. During inpatient care ultrasound was performed twice a week in asymptomatic recipients or in the case of local medical conditions. After hospital discharge regular follow-up examinations (including ultrasound) were done in our nephrological transplant outpatient clinic at 1, 2, 3, 6, and 12 months post-transplant. Additionally, the recipients were regularly seen by their local nephrologist.

### Clinical data collection, definitions, and statistical analysis

Clinical data were collected from clinical records and the database for transplant recipients. Follow-up data were collected from the responsible nephrologists throughout Germany. Delayed graft function (DGF) was defined by the need of at least one dialysis treatment during the first postoperative week. All complications occurring within the first year after KTx are included in the analysis. Lymphocele formation was defined as newly occurring perirenal fluid collection of more than 100 ml following transplantation determined by ultrasound examination after removal of wound drainage. In all cases, a diagnostic puncture to distinguish a potential urinoma or hematoma from lymphoceles was carried out. Bleeding/hematoma were specified by the need of reoperation or a sonographically proven perirenal hematoma.

Data are expressed as a median ± 95% confidence interval where applicable. Comparisons of groups for calculated data were performed using the Mann-Whitney U test and for categorical data using 2-tailed Fisher’s exact test. Patient and graft survival are depicted as Kaplan-Meier graphs and compared with Log rank. RBCT and weight of the removed polycystic kidney are depicted as Scatter plot correlation. The statistical analysis was carried out using IBM SPSS statistics, Version 23.0 and MedCalc® V 19.0.7 (MedCalc Software, www.medcalc.org). Statistical significance was defined as a *p* value of < 0.05.

## Results

### Match criteria and preoperative baseline characteristics of study population

Apart from the matching criteria, there were no other significant differences in donor or recipient characteristics such as BMI, donor gender composition, and ASA status. Only recipient gender composition differed significantly. In ADPKD, there was an equal distribution between male and female gender and in the control group there were significantly more female recipients (Table 1).

### Intra- and postoperative course and medical complications

The immunosuppressive regimen initially comprised a maintenance therapy with CNI, MPA, and steroids in almost all recipients. Two thirds of the recipients received an additional induction therapy, i.e., IL2-RA (59.1% ADPKD vs. 53.9% control) or in immunized recipients ATG (6.2% ADPKD vs. 5.7% control). In ABOi KTx recipients, rituximab and immunoadsorption were routinely applied [10, 11]. The operative time in ADPKD patients was 30 min longer on average (ADPKD 169 min; 95% CI 159.8–175.6 min vs. control 139 min; 95% CI 131.4–145.0 min; *p* < 0.0001). In the ADPKD group, anastomosis time (26 min) was slightly, yet significantly shorter compared with 29 min in the control group (95% CI 27.0–30.0 min; *p* = 0.0186). The mean weight of the removed polycystic kidney was 1.88 ± 0.16 kg (range 0.19–8.85 kg; *n* = 193). The weight corresponds approximately to the volume of the specimen. Pathological examination detected two previously unsuspected renal cell carcinomas. DGF, hospital and intensive care unit (ICU) stay were comparable between both groups. About a quarter of the patients in each group required a renal biopsy for clinically suspected rejection within the first 30 days after transplantation. The incidence of acute rejection (8.3% ADPKD vs. 11.9% control *p* = 0.3109) was comparable in both groups. CMV infections occurred in 2.6% (ADPKD) vs. 6.2% (control; *p* = 0.1346) during inpatient treatment, postoperative pneumonia in 2.6% (ADPKD) vs. 4.7% (control; *p* = 0.4152). UTIs were observed significantly more often in the ADPKD group (40.4%) compared with the control group (29.0%; *p* = 0.0246; Table 2).

**Table 2:** Intra- and postoperative course and medical complications

	ADPKD group (*n* = 193)	Matched control group (*n* = 193)	*p* value
*Initial Immunosuppression*			
CNI/MPA/steroids (in %)	191 (99.0%)	189 (97.9%)	0.6850
IL2-RA (in %)	114 (59.1%)	104 (53.9%)	0.3555
ATG (in %)	12 (6.2%)	11 (5.7%)	1.0000
Rituximab/immunoadsorption (in %)	22 (11.4%)	23 (11.9%)	1.0000
*Intra- and perioperative course*			
Operative time (in min.)	169.0 (95% CI 159.8–175.6)	139.0 (95% CI 131.4–145.0)	< 0.0001
Anastomosis time (in min.)	26.0 (95% CI 25.0–29.0)	29.0 (95% CI 27.0–30.0)	0.0186
Weight of polycystic kidney (in g)	1879.8 ± 162.9		
Malignant specimen (in %)	2 (1.0%)		
Delayed graft function (in %)	5 (2.6%)	4 (2.1%)	1.0000
Hospital stay (in d)	19.0 (95% CI 18.0–20.0)	18.0 (95% CI 17.0–19.0)	0.1292
Intensive care unit stay (in d)	0.0 (95% CI 0.0–0.0)	0.0 (95% CI 0.0–0.0)	0.9512
*Immunological and infectious course*			
Renal biopsies within 30 days (in %)	50 (25.9%)	45 (23.3%)	0.6366
Biopsy proven acute rejections ≤ 30days (in %)	16 (8.3%)	23 (11.9%)	0.3109
Inpatient CMV infections > 1000 copies (in %)	5 (2.6%)	12 (6.2%)	0.1346
Inpatient UTI (in %)	78 (40.4%)	56 (29.0%)	0.0246
Postoperative pneumonia (in %)	5 (2.6%)	9 (4.7%)	0.4152

Data are *n* (%) or median ± 95% CI. *p* values are estimated with Mann-Whitney U test for calculated data, and 2-tailed Fisher’s exact test for categorical data. Weight of polycystic kidney is expressed in mean ± SD

### Incidence of surgical complications and risk of reoperation within 30 days after KTx

There was no significant difference in the incidence of early vascular and urologic complications, lymphoceles, and wound healing disturbances. Severe non-renal-related surgical complications requiring reoperation were rare and comprised an ileus (ADPKD), an occlusion of the external iliac artery with critical limb ischemia (control). Thirty-day mortality was comparable in both groups (0.5% in ADPKD vs. 0.0% in control; *p* = 1.0000). In general, the reoperation rates (30 days and 1 year) were similar (Table 3). The incidence of postoperative hematoma/bleeding (ADPKD 8.3%; control 9.3%; *p* = 0.8578) was similar as well as the rate of reoperation within 30 days due to hematoma/bleeding (ADPKD 5.7%; control 8.3%; *p* = 0.4253). However, intraoperative RBCTs were required significantly more often in the ADPKD group (22.8%) compared with control (6.7%; *p* = 0.0001). After surgery, the number of patients, who needed more than one RBCT between postoperative day 3 and 7, was comparable in both groups (Table 3). Postoperative complications were categorized using the Clavien-Dindo Classification. The significant difference found between grade I and II was all attributable to a higher rate of postoperative UTIs in ADPKD patients (Table 4).

**Table 3:** Surgical morbidity and mortality within first year after KTx

		ADPKD group (*n* = 193)	Matched control group (*n* = 193)	*p* value
*Vascular complications*				
Venous thrombosis (in %)		2 (1.0%)	0 (0.0%)	0.4987
Severe arterial stenosis (in %)		2 (1.0%)	3 (1.6%)	1.0000
Graft nephrectomy ≤ 30days (in %)		2 (50.0%)	2 (66.7%)	1.0000
*Bleeding/hematoma*				
Intraoperative RBCT (in %)		44 (22.8%)	13 (6.7%)	0.0001
Postoperative bleeding/hematoma		16 (8.3%)	18 (9.3%)	0.8578
Postoperative RBCT (> 1 RBCT) post-OP day < 3 days		88 (45.6%)	43 (22.3%)	0.0001
Postoperative RBCT (> 1 RBCT) post-OP day 3–7 days		59 (30.6%)	48 (24.9%)	0.2554
*Urologic complications*				
Urinary obstruction (in %)		8 (4.2%)	11 (5.7%)	0.6391
Urinary leakage (in %)		5 (2.6%)	3 (1.6%)	0.7237
*Perirenal fluid collections*				
Lymphoceles (in %)		20 (10.4%)	26 (13.5%)	0.4324
*Wound healing*				
Wound infection (in %)		7 (3.6%)	13 (6.7%)	0.2504
Dehiscent fascia (in %)		2 (1.0%)	5 (2.6%)	0.4488
Hernia (in %)		2 (1.0%)	3 (1.6%)	1.0000
*Severe other surgical complications*				
Other surgical complications with reoperation ≤ 30 days (in %)		1 (0.5%)	1 (0.5%)	1.0000
*Reoperation rate and mortality*				
Reoperation	≤ 30 days (in %) Within first year (in %)	24 (12.4%) 48 (24.9%)	33 (17.1%) 55 (28.5%)	0.2509 0.4900
Mortality ≤ 30 days (in %)		1 (0.5%)	0 (0.0%)	1.0000

Data are *n* (%). *p* values are estimated with 2-tailed Fisher’s exact test for categorical data

**Table 4:** Clavien-Dindo classification

	ADPKD-group (*n* = 193)	Matched control group (*n* = 193)	*p* value
I	22 (11.4%)	39 (20.2%)	0.0250
II	132 (68.4%)	109 (56.5%)	0.0206
IIIa	3 (1.6%)	4 (2.1%)	1.0000
IIIb	27 (13.4%)	36 (18.7%)	0.2705
IVa	8 (4.2%)	5 (2.6%)	0.5744
IVb	0 (0.0%)	0 (0.0%)	1.0000
V	1 (0.5%)	0 (0.0%)	1.0000

Data are *n* (%). *p* values are estimated 2-tailed Fisher’s exact test for categorical data

### Short-term patient and death-censored graft survival

**Fig. 2:**
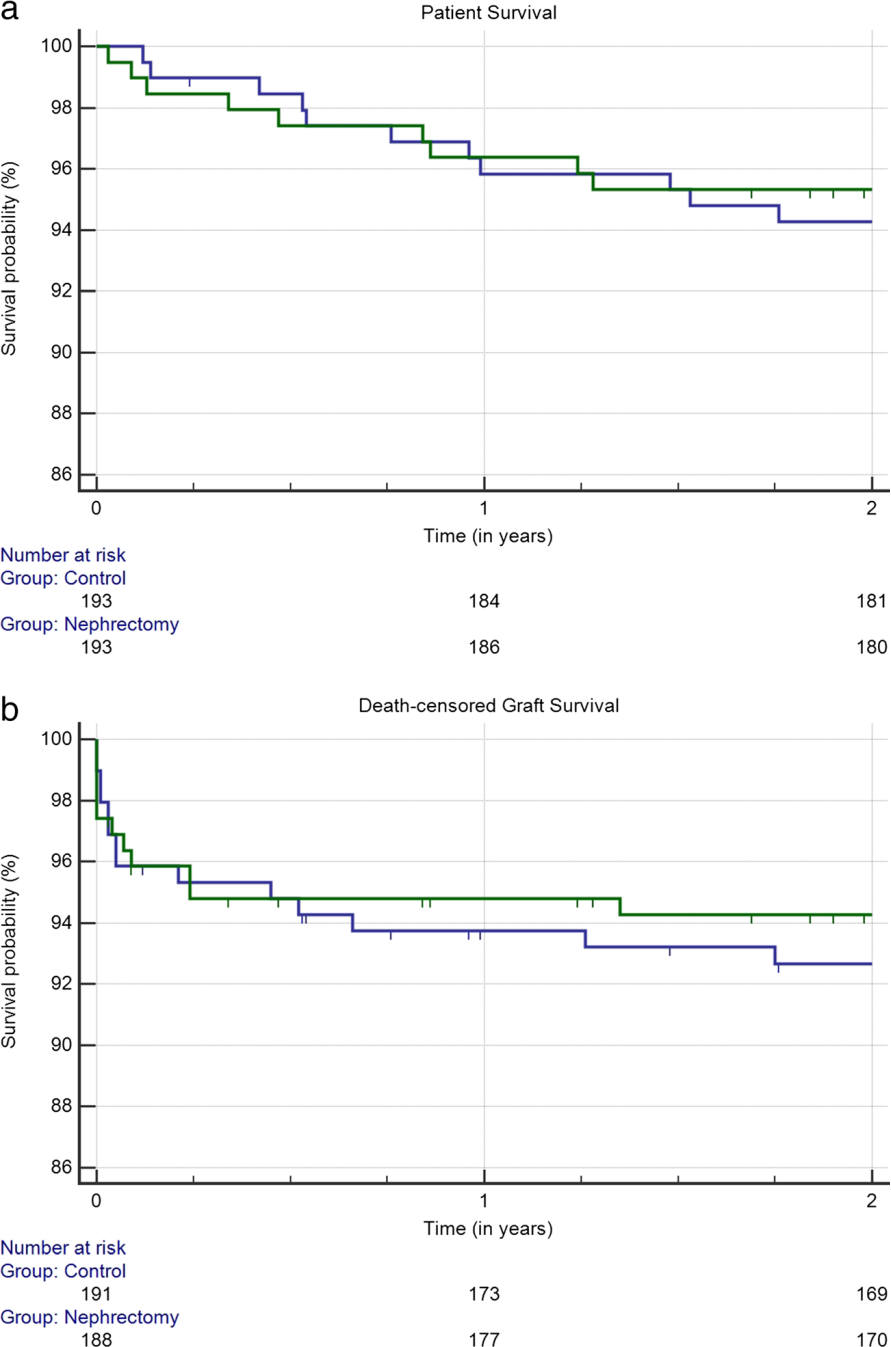
Kaplan-Maier survival probability of ADPKD group (blue) and control group (green). (a) Patient survival (*p* = 0.6537); (b) death-censored graft survival (*p* = 0.5479)

Simultaneous native nephrectomy in ADPKD group had no negative impact on short-term survival rates. Patient survival rates (ADPKD vs. control) after 1 year (96.4% vs. 95.8%) and after 2 years (95.3% vs. 94.3%) were comparable (*p* = 0.6537;Fig. 2a). Death-censored graft survival (ADPKD vs. control) was 94.8% vs. 93.7% at 1 year and 94.3% vs. 92.7% at 2 years (*p* = 0.5479; Fig. 2b).

### Subgroup analysis of ADPKD recipients

**Fig. 3:**
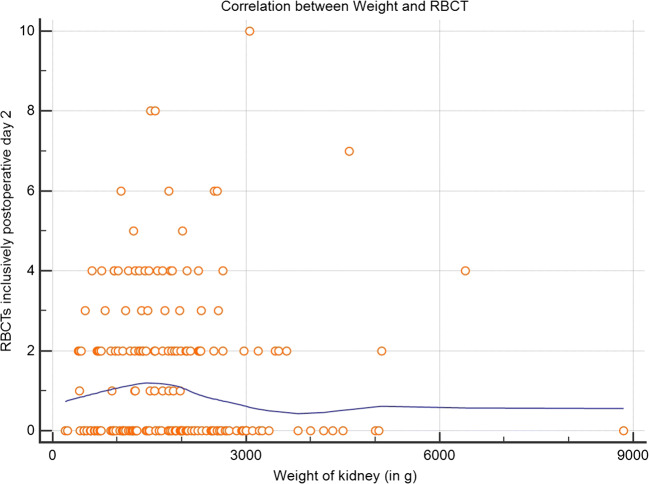
Correlation of red blood cell transfusions and weight of removed polycystic kidney LOESS smoothing span 80%

The mean weights of the polycystic kidneys were 1879.8 ± 162.9 g, ranging from 193 to 8850 g and were roughly equivalent to volume. The number of RBCTs required at the day of transplantation and within the first two postoperative days did not correlate with the weight/size of the removed native kidney (*p* = 0.9032; Fig. 3).

### Course of disease regarding the asymptomatic contralateral polycystic kidney

To investigate the fate of the contralateral kidney under immunosuppression, we divided ADPKD recipients into three subgroups: (a) pre-transplant contralateral nephrectomy was necessary due to a symptomatic course (*n* = 38; 19.7%; no existing native polycystic kidney after transplantation), (b) no need for post-transplant contralateral nephrectomy (109 patients; 56.5%; contralateral kidney still in-situ), and (c) post-transplant contralateral nephrectomy was necessary due to a symptomatic course under immunosuppression (*n* = 46; 23.8%). A post-transplant nephrectomy of the contralateral kidney in groups (b) and (c) (*n* = 155) was necessary in 29.7% (*n* = 46) of the recipients at different points in time and due to various reasons. Compared with recipients in group a, the percentage of infections leading to contralateral nephrectomy was significantly increased in group (c) (7.9% vs. 34.8%; *p* = 0.0038; Table 5). On the contrary, the rate of UTIs during the inpatient care after transplantation was similar for all three groups (42.1% for group a, 40.4% for group (b), and 39.1% for group (c)).

**Table 5:** Indication for nephrectomy prior and after KTx

	Prior KTx (group a) (*n* = 38)	Post KTx (group c) (*n* = 46)	*p* value
Cyst infection/UTI (in %)	3 (7.9%)	16 (34.8%)	0.0038
Bleeding/hematuria (in %)	3 (7.9%)	3 (6.9%)	1.0000
Pain (in %)	5 (13.2%)	6 (13.1%)	1.0000
Renal mass/digestive problems (in %)	27 (71.1%)	17 (37.0%)	0.0023
Renal cell carcinoma in ipsilateral kidney (in %)	0 (0.0%)	2 (4.4%)	0.4986
Suspicion for renal cell carcinoma (in %)	0 (0.0%)	1 (2.2%)	1.0000
Other (in %)	0 (0.0%)	1 (2.2%)	1.0000

Data are *n* (%). *p* values are estimated 2-tailed Fisher’s exact test for categorical data

The median size of the simultaneously removed polycystic kidney during KTx in group (b) was 1581 g (CI 95%, 1347–1795 g). It therefore was significantly lower than in groups (a) (2080 g; CI 95% 1460–2562 g; *p* = 0.0224) and (c) (2049 g CI 95% 1561–2388 g; *p* = 0.0101). The size of the polycystic kidney in groups (a) and (c) were not different (*p* = 0.9857).

## Discussion

Our results clearly demonstrate that per protocol simultaneous ipsilateral nephrectomy of polycystic kidney in ADPKD KTx recipients is a safe procedure, which does not increase mortality or morbidity in our center. There is only a slightly increased need of RBCTs during the early postoperative course, possibly reflecting a larger surgical field. Short-term patient and graft survival rates in ADPKD KTx recipients with simultaneous native nephrectomy were similar compared with KTx recipients of the control group. Therefore in our opinion, the increased duration of surgery, approximately 30 min, appears to be well justified.

Surprisingly, the observed higher rate of inpatient UTIs were unrelated to the remaining contralateral polycystic native kidney (as a possible source of recurrent infections under immunosuppression) compared with patients which had the contralateral kidney removed pre-transplant. A plausible explanation might be a subliminal bacterial colonization of the urinary tract in the ADPKD patients which possibly changes into a virulent state under the required immunosuppressive therapeutic regimen originating from the removed organ during surgery. In the long-term, only a third of the recipients with a remaining contralateral polycystic kidney required a nephrectomy after KTx.

In a review in 2017, an algorithm for the management of nephrectomy in ADPKD KTx recipients on dialysis was published. The program suggests that no pre-transplant nephrectomy is indicated in asymptomatic patients. In symptomatic patients, residual diuresis ought to be included as an additional parameter in the decision making [12]. In recipients with residual diuresis, pre-transplant unilateral nephrectomy should be favored. In living donation, the simultaneous procedure is preferred [12]. In the absence of diuresis, the algorithm recommends a bilateral pre-transplant or simultaneous nephrectomy (if living donor KTx). Independently of the recommendation, in symptomatic patients not yet on dialysis, the indication for nephrectomy should be set very carefully, being aware, that these patients are most likely on dialysis immediately afterward.

In asymptomatic patients, however, the indication and timing of nephrectomy awaiting kidney transplantation remains controversial. Generally, applicable guidelines do not exist. Thus, the approach is often center-specific and based on personal training and experience. The decision in favor of nephrectomy of the polycystic kidney is mostly based on space considerations and performed in 20−33.1% of the patients [3, 13–15]. Results of two studies comparing recipients of pre-transplant and/or simultaneous nephrectomy with recipients receiving no nephrectomy are inconclusive [16, 17]. Sulikowski et al. reported that transplant recipients without nephrectomy experienced UTIs in 42.9% and cyst infections in 38%, leading to graft loss in 19.1% of cases. Based on the latter data, pre-transplant or simultaneous bilateral nephrectomy was recommended [16]. Since simultaneous ipsilateral nephrectomy was carried out per protocol at our center, our control group consisted of non-ADPKD KTx recipients only. Therefore, we suppose, that the higher rate of UTIs is not caused by surgical impact of the simultaneous nephrectomy, but rather by the ADPKD as underlying kidney disease itself. In contrast, renal-related complications with 34% cyst-related infections appeared to be more common in patients without nephrectomy compared with 20% in recipients with nephrectomy. However, this difference was not statistically significant and therefore routine nephrectomy concurrently is discussed controversial [17]. Yet, with respect to the indication, the results are variable and clear decision guidance cannot be deduced from these data at present. Therefore, decisions usually are based on the subjective evaluation and experience of the attending surgeon or as in this study by protocol.

Focusing on surgical complications, a benefit favoring a certain time-point (pre- or post-transplant) did not emerge [15, 18, 19]. Of note, residual diuresis and pre-transplant hemoglobin are significantly lower in patients receiving pre-transplant nephrectomy. This procedure should be reserved for highly symptomatic patients [19]. Even simultaneous uni- or bilateral procedures did not yield less favorable results with the reasonable exception of an extended operative time and more intraoperative infusions, plasma, and blood transfusions [14, 18, 20–22]. Compared with renal KTx recipients without nephrectomy, perioperative mortality, morbidity, patient, and graft survival rates are similar in simultaneous unilateral procedures [19]. For simultaneous bilateral procedures, blood loss was similar as well, tested against recipients receiving a staged surgery, including blood loss during pre-transplant bilateral nephrectomy and subsequent transplantation [23]. Ferrandiz et al. reported higher risk of donor-specific antibodies after early RBCTs [24]. Especially, recipients receiving pre- and post-transplant RBCTs are at risk for antibody-mediated rejection [25]. Therefore, simultaneous procedure could be advantageous to staged procedures.

Data comparing the extent of nephrectomy (unilateral vs. sandwich vs. bilateral) are scarce. Kirkman et al. reported the need of ICU admission in a small series comparing unilateral and bilateral nephrectomies before and after transplantation. With 25% in the unilateral and 20% in the bilateral group, severe complications were similar. The 3 patients treated with sandwich technique experienced no severe complication, but based on the low case number, no valid conclusion could be derived [26]. In contrast, a significantly higher rate of renal vascular thrombosis (4.4% vs. 0%) is presented in simultaneous bilateral procedures compared with pre-transplant nephrectomy [23]. In all other publications, there is a clear preference for one of the procedures. Kramer et al. emphasize the safety of simultaneous bilateral nephrectomy in 20 cases [27]. In 2013, we published the results of the first 100 consecutive KTx recipients receiving a simultaneous unilateral nephrectomy [4]. The rate of surgical complications was low and the technique safe. These patients are included in the present study. According to existing data then, both uni- and bilateral procedures are safe and can be individually adapted to the prevailing situation and preference.

Concerning the ideal time-point of nephrectomy, pre-transplant, concurrent, and post-transplant procedures were compared in several past retrospective studies. With regard to residual renal diuresis, Fuller et al. reported that pre-transplant bilateral nephrectomy is less desirable [18]. However, another group preferred bilateral nephrectomy in symptomatic patients, either performed simultaneously, if living donation was realized, or pre-transplant in patients on the waiting list for cadaveric KTx [28]. With respect to patient and graft survival and complications, no differences were reported [28].

Based on a few cases, a report evaluated the safety of laparoscopic procedures in pre- and post-transplant native nephrectomy. Surgical complication rates are similar to open procedures [15, 24]. In respect to postoperative pain and hospital stay, laparoscopic techniques have an advantage [15, 29]. In laparoscopically treated patients, the renal mass is significantly smaller whereas the mean operative time of native nephrectomy is similar (172.5–220.0 min) [29, 30]. With respect to laparoscopically simultaneous native nephrectomy, no published data are available. One ought to consider, however, the consequences of prolonged cold ischemic time.

The incidence of renal cell carcinoma was reported with 1.3% [31] and therefore similar to the above presented data. Special attention on hematuria, often being the first evidence for renal cell carcinoma is warranted. In an earlier report from our center, the prevalence of renal cell carcinoma was reported with 5% of 240 renal specimens of ADPKD patients [32].

In symptomatic recipients, the preferable approach appears rather obvious. However, in asymptomatic ADPKD recipients, the decision is more challenging. On the one hand, it is doubtful if a major extension of the KTx transplant procedure is justified, knowing that it might have no benefit to many recipients. On the other hand, in recipients without nephrectomy, complications of severe and even lethal infections have been described during the post-transplant course under immunosuppression. Repeatedly, the safety of simultaneous nephrectomy, particularly with respect to surgical complications, as well as patient and graft survival, was demonstrated earlier and is strongly bolstered by this study [14, 18, 20–22]. The decision for a simultaneous nephrectomy should be handled broadmindedly, rather than to endanger patient and graft after KTx. Considering the rate of early renal vascular complications during simultaneous bilateral nephrectomy and the fact that only approximately 30% of the recipients need post-transplant contralateral nephrectomy, clearly the unilateral approach should be favored.

### Limitations of the study

We are aware that the decision to perform simultaneous ipsilateral nephrectomy in all ADPKD patients at the end of the 1990s at our center was only based on favorable clinical courses from the years before. Owing to an experienced surgical team which could provide nephrectomy and transplantation by one surgeon at any time, a randomization of patients was not undertaken back then. Therefore, the best way to compare results without changing the protocol was to do a single-center matched pair analysis with non-ADPKD patients transplanted by the same surgical team during the same time period.

## Conclusions

Simultaneous unilateral nephrectomy in ADPKD KTx recipients in this setting was a safe procedure. The incidence of postoperative morbidity and mortality was not increased compared with non-ADPKD recipients receiving KTx alone, except for a more frequent need of RBCTs and a higher rate of early postoperative UTIs. With respect to possible severe infectious complications under immunosuppression, even in asymptomatic ADPKD KTx recipients’, we conclude that the indication for unilateral native nephrectomy should be set broadmindedly. In addition, the number of total operations can be reduced and residual diuresis preserved up until transplantation. In living donor KTx recipients—which comprises a large group at our center—this technique enables true preemptive transplantation.

## Abbreviations

ABOc: blood group compatible
ABOi: blood group incompatible
ADPKD: autosomal-dominant polycystic kidney disease
ASA: American Society of Anesthesiologists
ATG: anti-thymocyte-globulin
BMI: body mass index
CI: confidence interval
CMV: cytomegalovirus
CNI: calcineurin-inhibitor
DGF: delayed graft function
DJ: double-J
ESRD: end-stage renal disease
ICU: intensive care unit
IL2-RA: interleukin-2-receptor antagonist
MPA: mycophenolic acid
KTx: kidney transplantation
PRA: panel reactive antibody
RBCT: red blood cell transfusion
UTI: urinary tract infection
